# ﻿Revision of the genus *Colasia* Koch, 1965 (= *Belousovia* Medvedev, 2007, syn. nov.) (Coleoptera, Tenebrionidae, Blaptini)

**DOI:** 10.3897/zookeys.1161.97440

**Published:** 2023-05-11

**Authors:** Xing-Long Bai, Jing-Ze Liu, Guo-Dong Ren

**Affiliations:** 1 Hebei Key Laboratory of Animal Physiology, Biochemistry and Molecular Biology, College of Life Sciences, Hebei Normal University, Shijiazhuang, Hebei 050024, China Hebei Normal University Shijiazhuang China; 2 The Key Laboratory of Zoological Systematics and Application, School of Life Sciences, Institute of Life Science and Green Development, Hebei University, Baoding, Hebei 071002, China Hebei University Baoding China

**Keywords:** Blaptinae, lectotype, new combinations, new species, new synonymy

## Abstract

The relationship between the genera *Colasia* Koch, 1965 and *Belousovia* Medvedev, 2007 within the tribe Blaptini is discussed, and a new synonymy is proposed: *Belousovia* Medvedev, 2007, **syn. nov.** of *Colasia* Koch, 1965. As a result, three new combinations are established: *Colasiahelenae* (Medvedev, 2007), **comb. nov.**, *C.kabakiintermedia* (Medvedev, 2007), **comb. nov.**, and *C.kabakikabaki* (Medvedev, 2007), **comb. nov.***Colasiaakisoides* Koch, 1965 is redescribed, and a lectotype is designated. Three new species of the genus *Colasia* are described and illustrated from China: *C.bijica***sp. nov.** (Guizhou), *C.medvedevi***sp. nov.** (Yunnan), and *C.pilosa***sp. nov.** (Yunnan). A distribution map and a key to species of the revised genus *Colasia* are presented.

## ﻿Introduction

The genera *Colasia* Koch, 1965 and *Belousovia* Medvedev, 2007 belong to the tribe Blaptini Leach, 1815 (Tenebrionidae, Blaptinae) (Koch 1965; Medvedev 2007; Kamiński et al. 2021). The genus *Colasia*, dedicated to M.G. Colas (Mr. Guy Colas 1902–1993; Dr. Christophe Hervé, pers. comm., March 2023), is represented only by the type species *C.akisoides* Koch, 1965 described from Chongqing, China. According to the original description, *Colasia* is similar to *Tagonoides* Fairmaire, 1886 as both have granulated elytra. Moreover, to a certain extent, *Colasiaakisoides* is also somewhat similar to *Asidoblapsglyptoptera* Fairmaire, 1886, also by the granules on the elytra (Koch 1965).

A new genus *Montagona* Medvedev, 1998 was established and compared with *Colasia* and the three other genera. The tribe Blaptini was subdivided into two subtribes by Skopin (1960) but later into five subtribes by Medvedev (2001), and *Colasia* and another ten genera were classified within the subtribe Gnaptorinina Medvedev, 2001. Based on the ovipositor structure, this subtribe Gnaptorinina was further divided into three subgroups (Medvedev and Merkl 2003):

1. *Gnaptorina* Reitter, 1887, *Itagonia* Reitter, 1887, *Montagona*, and *Tagonoides*, characterized by apically cuneate and narrowed ovipositorial lobes;
2. *Agnaptoria* Reitter, 1887, *Asidoblaps* Fairmaire, 1886, *Nepalindia* Medvedev, 1998, and probably also *Sintagona* Medvedev, 1998 (female unknown), characterized by apical margin of ovipositorial lobes obliquely truncate;
3. *Colasia*, and *Viettagona*, characterized by apically rounded ovipositorial lobes.

The genus *Belousovia*, named for Igor Alexandrowich Belousov, is represented by one species and two subspecies described from western Yunnan, China: *B.helenae* Medvedev, 2007, *B.kabakiintermedia* Medvedev, 2007, and *B.kabakikabaki* Medvedev, 2007. According to the original description (Medvedev 2007), *Belousovia* closely resembles *Colasia* (based on the examination of *C.medvedevi* sp. nov., erroneously determined by N. Skopin and G. Medvedev as *C.akisioides*) in the structure of aedeagus, spiculum gastrale, ovipositor, and elytra, but clearly differs from *Colasia* by the structure of legs, female genital tubes, and head capsule. The first and the most specific character, male legs: in *Belousovia* species, the ventral surface of pro- and mesotarsomeres I–IV, and metatarsomeres I–III with long and dense hairy tuft, the apical part of the metatibiae with a row of dense setae on the inner side (e.g., *B.kabakikabaki* Medvedev, 2007: figs 116, 117); in *Colasia* species, the ventral surface of tarsal segments without hairy brush or tuft (from Koch 1965), only very short and strong setae present, metatibiae without a row of setae on the inner side. The second character, head capsule: the labrum and apical maxillary palpomere are covered with long setae in *Belousovia* species, but are short in *Colasia* species; eyes short and distinctly arcuately projecting outwards in *Belousovia* species, but absolutely flat in *Colasia* species. The third character, appearance: the pronotum is obviously heart-shaped in *Colasia* species, which never occurs in *Belousovia* species; the body is coal-black in *Colasia* species, but appears reddish in strong illumination in *Belousovia* species, especially on the humeral carinae and declivity of the elytra. The last character, female genital tubes: in *Colasia* species, bases of first and second reservoirs and base of spermathecal sphincter diverge from one point; in *Belousovia* species, the base of the first reservoir is separated from the place of divergence of the bases of the second reservoir and spermathecal sphincter by a very long duct. However, the boundary between the genera *Belousovia* and *Colasia* becomes blurred with the examination of additional materials from western Guizhou, and eastern, central and western Yunnan, China.

This study aims to investigate the taxonomic status of the genera *Colasia* and *Belousovia*. Additionally, a redescription of *C.akisoides* and descriptions of three new Chinese species are provided.

## ﻿Material and method

The specimens were examined and dissected under a Nikon SMZ800 microscope, and photographs were taken using Canon EOS 5DSR camera and processed by Adobe Photoshop 2021. The distribution map was made by QGIS and processed by Adobe Photoshop 2021. Aedeagi was detached from the body with insect pins, then glued to separate cards and pinned under the specimens. A single slash (/) separates data of different lines on a label, a double slash (//) separates data of different labels, authors’ remarks are enclosed in brackets “ []”.

Specimens examined in this study are deposited at the following institutes and collections:

**CTLH** private collection of Tian-Long HE, Huainan, China;

**HBUM**Hebei University Museum, Baoding, China;

**HNHM**Hungarian Natural History Museum, Budapest, Hungary;

**MYNU** Invertebrate Collection of Mianyang Normal University, Sichuan, China;

**ZIN**Zoological Institute of Russian Academy of Sciences, St.-Petersburg, Russia.

## ﻿Taxonomic accounts

### ﻿Genus Colasia

Taxon classificationAnimaliaColeopteraTenebrionidae

Koch, 1965

C9735091-90BA-5A7F-87F7-B8C76CAFD8B8

Colasia Koch, 1965: 131; Medvedev 2001: 95; Löbl et al. 2008: 231; Ren et al. 2016: 333; Nabozhenko and Chigray 2020: 285; Bouchard et al. 2021: 44. Type species: Colasiaakisoides Koch, 1965, by monotypy.
Belousovia Medvedev, 2007: 157; Ren et al. 2016: 328; Nabozhenko and Chigray 2020: 285; Bouchard et al. 2021: 44. Syn. nov. Type species: Belousoviahelenae Medvedev, 2007, by original designation.

#### Remarks.

After the examination of types of the genera *Belousovia* and *Colasia*, and also additional materials, we propose the genus *Belousovia* Medvedev, 2007 as a junior synonym of the genus *Colasia* Koch, 1965.

Firstly, in male legs: after the re-examination of the types of *Colasiaakisoides*, apical part of metatibiae with a row of setae on the inner side (Fig. 5G, G’; not mentioned in the original description by Koch 1965), although less dense than *Belousovia* species (Fig. 19G, G’), but dense enough compared to materials from Western Guizhou, and Western Yunnan (Figs 6G, G’, 20; a few setae present only); ventral surface of tarsal segments with hairy tuft at apex (Fig. 5E, F, H). As emphasized by Medvedev (2007), the degree of development of the setae on ventral surface of pro- and mesotarsomeres of the male, and the setae on the inner side of tibiae steadily characterizes morphological distinctiveness of separate genera in the subtribe Gnaptorinina. However, Medvedev is sometimes a little over-dependent on the degree of development of the setae (Medvedev 2009), and this has been questioned by the molecular evidence (Li et al. 2021). Moreover, acquired activities can also damage setae. Thus, the first and the most important evidence presented by Medvedev (2007) is untenable. Secondly, in head capsule, and in appearance: setae on labrum and maxillary palpi, size and shape of eyes, shape of pronotum, and coloration of body are common characters, they may be distinct in separate genera, but are also variable between different species within a genus. Besides, the author is a bit exaggerates the differences between the genera *Belousovia* and *Colasia*, as the pictures presented in the publication (Medvedev 2007). Lastly, the female genital tubes can indeed be used to separate genera, as the author used in the diagnosis (Medvedev 2007), but is not a decisive character. Therefore, the last three distinctions observed by Medvedev are perhaps not enough to distinguish the genera *Belousovia* and *Colasia*.

### ﻿ Colasia akisoides

Taxon classificationAnimaliaColeopteraTenebrionidae

Koch, 1965

00931D5E-4911-59E5-9428-7B05B0481D94

Figs 1–4 , 5 , 28 , 29 , 33

Colasia akisoides Koch, 1965: 131; Medvedev 2001: 95; Löbl et al. 2008: 231; Ren et al. 2016: 333; Nabozhenko and Chigray 2020: 285.

#### Type material.

**Figures 1–4.**
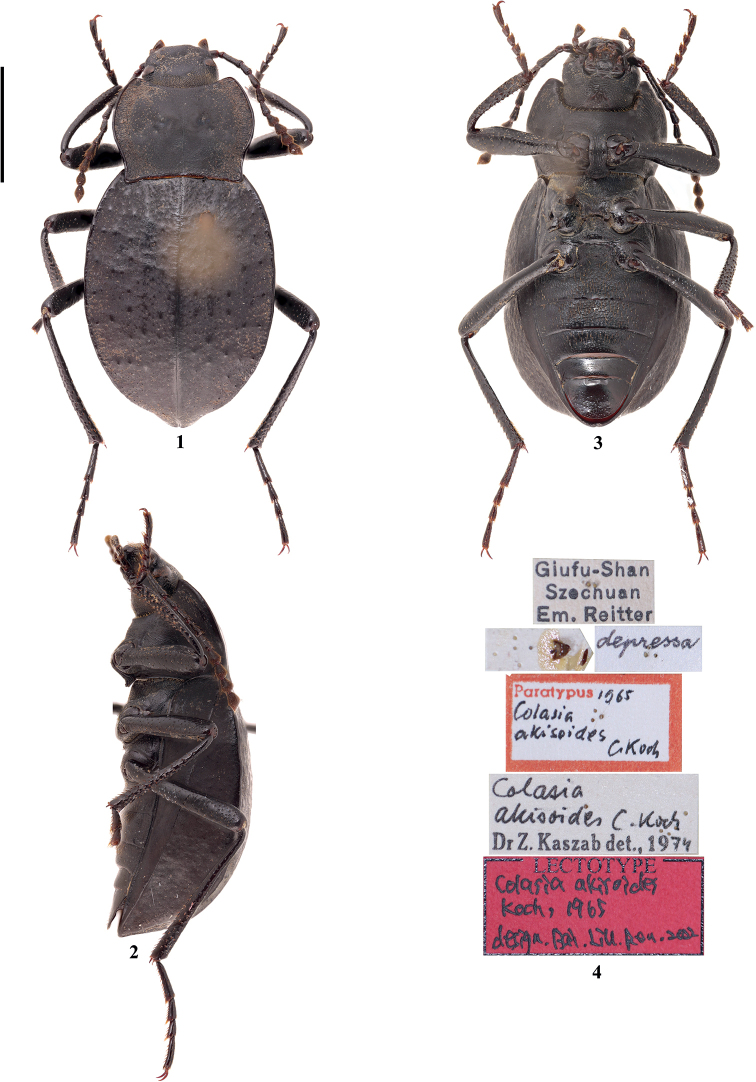
Habitus and type labels of *Colasiaakisoides* Koch **1–3** lectotype, male in **1** dorsal **2** lateral, and **3** ventral views **4** type labels. Scale bar: 3.5 mm.

***Lectotype*, designated here**: ♂ (HNHM), Giufu-Shan / Szechuan / Em. Reitter // *depressa* // Paratypus 1965 / *Colasiaakisoides* / C.Koch // *Colasia* / *akisoides* C.Koch / Dr Z. Kaszab det., 1974 // LECTOTYPE / *Colasiaakisoides* / Koch, 1965 / design. Bai, Liu, Ren, 2022 (Figs 1–4). ***Paralectotypes***: 1♀ (HNHM), ♀ // Giufu-Shan / Szechuan / Em. Reitter // *depressa* // Paratypus 1965 / *Colasiaakisoides* / C.Koch // Paralectotype / *Colasiaakisoides* / Koch, 1965 / design. Bai, Liu, Ren, 2022; 1♀ (HNHM), ♀ // Kinfushan / Prov. Szechuan / West-China IV/V 29 / Coll.,H.Becker // *depressa* // Paratypus 1965 / *Colasiaakisoides* / C.Koch // Paralectotype / *Colasiaakisoides* / Koch, 1965 / design. Bai, Liu, Ren, 2022.

#### Additional material.

**China**: 1♂ (HBUM), 2015.VII.10 / 1665 m / North Slope of Jinfo Shan, Nanchuan District, Chongqing / 29°02'34"N / leaf litter / 107°11'10"E / Ri-Xin JIANG leg. / Mianyang Normal University, MYNU; 2♀ (HBUM), 2022.VI.30-VII.3 / Shangding, North Slope of Jinfo Shan, Chongqing / 2000 m / Tian-Xuan GU leg. / Hebei University Museum.

**Figure 5.**
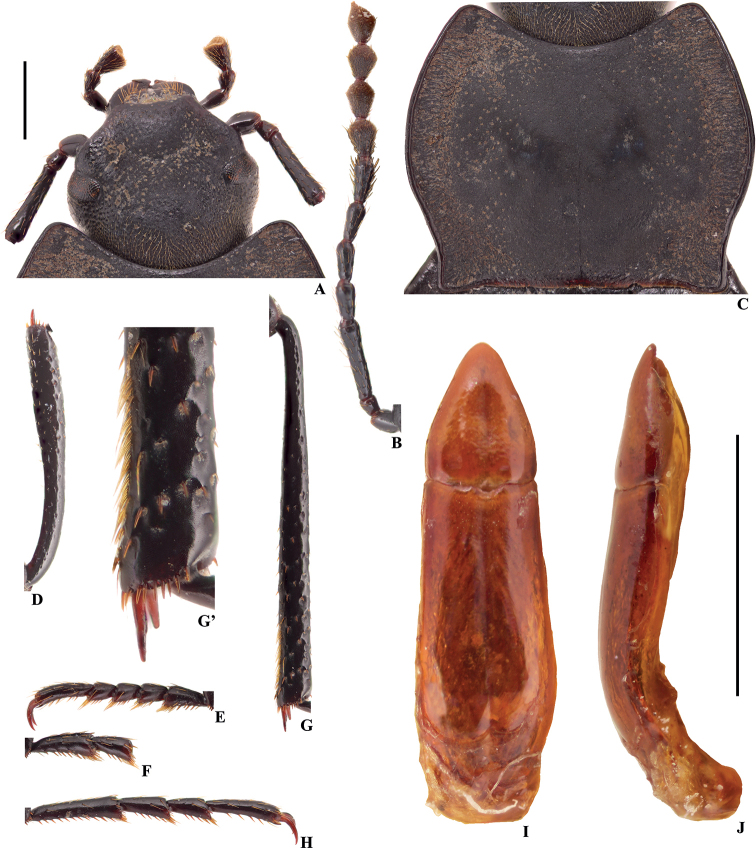
Characters of *Colasiaakisoides* Koch, male, lectotype **A** head **B** antenna **C** pronotum **D** protibia **E** protarsus **F** mesotarsus (mesotarsomeres III–V are missing) **G** metatibia **G**’ distal part of metatibia **H** metatarsus **I, J** aedeagus in dorsal and lateral view, respectively. Scale bars: 1.0 mm.

#### Distribution.

China: Chongqing.

#### Remarks.

This species was described based on the collection of Muséum national d’Histoire naturelle, Paris, France (Koch 1965), including ten specimens from China in the original description: “2♂♂ 2♀♀ <<Giufu-Shan, Szechuan>>! leg. Emm. REITTER; 1♂ 4♀♀, Kintushan, Prov. Szechuan, West-China>>! leg. BECKER; 1 ♀ <<Junan, Junan-fu>>!”.

In January 2017, the first author had a chance to visit the Muséum national d’Histoire naturelle, but no types of this species have been found. In May 2022, XB asked Dr. Antoine Mantilleri (curator for Coleoptera at the MNHN) for help in searching for the types of this species. No such specimens were identified. Fortunately, three syntypes (1♂, 2♀) of this species were founded in HNHM and lent us to study by late Dr. Ottó Merkl. Therefore, the male type deposited in HNHM is designated as the lectotype in this paper, and the remaining types becoming paralectotypes.

The identification of the female paralectotype from Junan-fu (most likely Kunming City), Junan (Yunnan) is in doubt. On the one hand, according to the distribution of all the species of the revised genus *Colasia*, it is unlikely to be *C.akisoides*. On the other hand, it was once identified as *Asidoblapsglyptoptera* by Gebien (Koch 1965). Recently, we have obtained some specimens from Kunming, Yunnan, and they were confirmed to be *Asidoblaps* sp. after the identification. These specimens superficially resembled representatives of *Colasia*, but were obviously different by aedeagal morphology. Thus, we speculate that the female paralectotype from Yunnan belongs to the *Asidoblaps* species.

Giufu-Shan and Kinfushan (sometimes erroneously spelled Kintushan) both refer to the current Jinfo Shan (Bezděk et al. 2015; Kataev and Liang 2015; Ge et al. 2021). Jinfo Shan is located in southern Chongqing, Chongqing once belonged to Sichuan Province, and now it is a municipality directly under the Central Government. Thus, the type locality of this species is Jinfo Shan, and the distribution of this species should be changed from Sichuan and Yunnan (Löbl et al. 2008; Ren et al. 2016; Nabozhenko and Chigray 2020) to Chongqing.

#### Redescription.

Body black, weakly shiny; legs shiny.

**Male. *Head*.** Apical maxillary palpomere triangular, covered with moderately dense and long setae. Anterior margin of labrum emarginate, lateral margins weakly arcuate. Anterior margin of epistoma emarginate; surface flat, matte, inconspicuous punctate. Frontoepistomal suture shallow and arcuate. Dorsal surface of head flat, matte, sparsely and finely granulated. Genal margins arcuately converging forwards, densely and shallowly punctures merged into short wrinkles. Emargination of outer margins of head above antennal base straight. Eyes transverse, not protruding beyond contour of head, distance between outer margins of eyes represent the widest of head; height 0.53 mm, width 0.17 mm from lateral view, respectively (height 3.1× width; 2.5× if the height and width rounded to one decimal place). Temples arcuately narrowing backwards, weakly granulated. Antennae slender and long, with the last segment reaching beyond pronotal base; basal part of antennomere I invisible in dorsal view; II–VII cylindrical, thicker at apex, II very short, III very long, V–VI equal in length, longer than IV and shorter than VII; VIII–X nearly spherical; XI sharped-oval.

***Prothorax*.** Pronotum cordiform, widest at middle, 1.5× wide as long, 1.8× wide as head, ratio of width at anterior margin to middle and base 7: 11: 8; anterior margin deeply emarginate, beaded laterally; lateral margins weakly “S” curved, entirely beaded and smooth; posterior margin straight at middle, beaded laterally; anterior angles rectangular and protruding forwards, posterior angles rectangular; surface matte, central convex, lateral sides weakly depressed along lateral margins, with shallowly and rounded depressions in sides of central, moderately depressed near posterior angles in sides of base, longitudinal median line (median depression in Medvedev 2007) smooth and inconspicuous; shallowly, sparsely and finely punctate in central part, near posterior margin, lateral margins, and lateral sides of anterior margin with wrinkly punctures, sparsely and finely granulated. Prothoracic hypomera depressed, densely and shallowly wrinkled in longitudinal, with sparse and tiny granules. Prosternal process sharply sloping downwards behind procoxae, apex blunt in lateral view.

**Figure 6.**
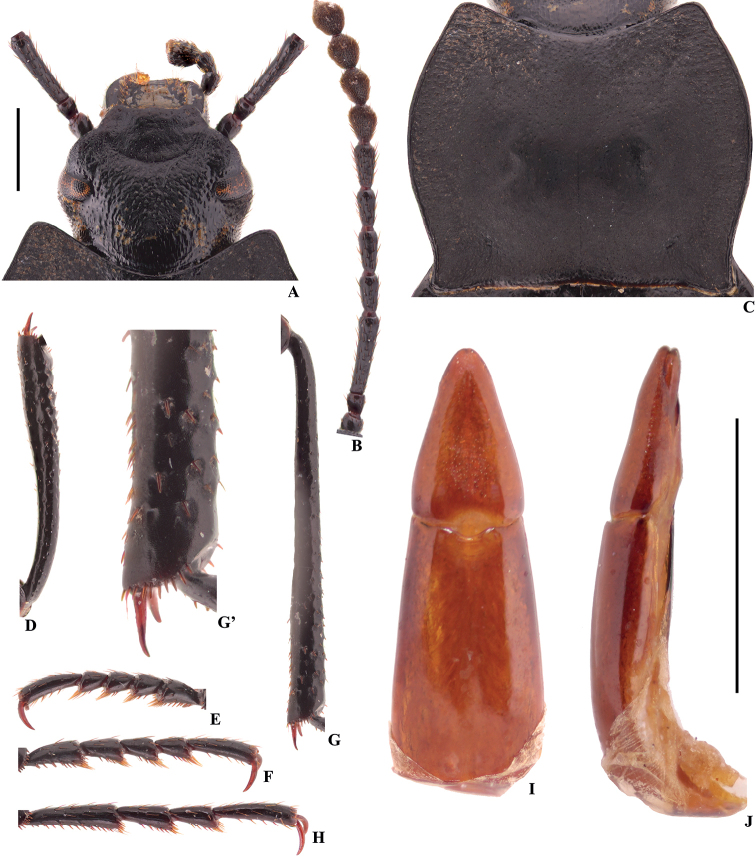
Characters of *Colasiabijica* sp. nov., male, holotype **A** head **B** antenna **C** pronotum **D** protibia **E** protarsus **F** mesotarsus **G** metatibia **G**’ distal part of metatibia **H** metatarsus **I, J** aedeagus in dorsal and lateral view, respectively. Scale bars: 1.0 mm.

***Pterothorax*.** Elytra oval, widest at middle, 1.3× long as wide, 1.3× wide as pronotum; base nearly as wide as pronotal base; dorsal surface matte, weakly convex, declivity sharply sloping downwards; humeral carinae smooth, with very sparse, smooth and large tubercles, sparse and fine granules, sparse, coarse and shallow wrinkles between humeral carinae; each elytron with two carinae (sensu Medvedev 2007) between suture and humeral carina, the second carina inconspicuous; declivity with sparse and short setae, slightly convex along suture; surface of epipleuron (sensu Kamiński et al. 2019) matte, edge relatively wide; pseudopleuron (outer part of elytra in Medvedev 2007) much wider than epipleuron, surface matte, with sparse and inconspicuous granules, edge thin and elevated, reaching sutural angle. Scutellum triangular, covered by pronotum.

***Abdomen*.** Apex of ventrite 1, and base of ventrite 2 flat in middle; ventrites 1, 2, and anterior part of ventrite 3 rough, with moderately dense and long setae, and inconspicuous granules, densely, shallowly, and finely wrinkled at sides; posterior part of ventrite 3 smooth, shallowly punctate; ventrite 4 smooth, shallowly and finely punctate; last ventrite smooth, with sparse, fine punctures and short setae, apical margin widely rounded.

***Legs*.** Slender and long. Femora claviform, mesofemora slightly longer than profemora, and shorter than metafemora. Protibiae weakly curved, distal part distinctly thick; mesotibiae and metatibiae straight, both gradually widened toward apex; distal part of metatibiae with a row of golden yellow hairy row on inner side. Ventral surface of pro- and mesotarsomeres I–IV, and metatarsomeres I–III with undeveloped hairy tuft at apex.

***Aedeagus*.** Length 1.8 mm, width 0.5 mm. Parameres length 0.5 mm, width 0.4 mm. Parameres relatively wide and short, widest at base, basal 1/4 parallel, and then narrowing toward apex nearly straight, distal part weakly curved to ventral side in lateral view.

**Female.** Antennae not reaching pronotal base; elytra wider and more convex; abdominal ventrites 1 and 2 convex; inner side of metatibiae without golden yellow setae; other characters similar to male.

#### Measurements.

Body length: 11.5–13.5 mm; width: ♂ 5.5–6.0 mm, ♀ 6.5–7.0 mm.

### ﻿ Colasia bijica sp. nov.

Taxon classificationAnimaliaColeopteraTenebrionidae

A4B80B2C-C22A-5C0E-81DA-35B136B06C95

https://zoobank.org/08015B8B-836B-4E4E-9BF0-A4EC716F786F

Figs 6 , 22 , 33

#### Type material.

***Holotype***: ♂ (HBUM), 2011-IV-24 / Jiucaiping, [Hezhang County, Bijie City], Guizhou / Wen-Bin JU leg.

#### Diagnosis.

This new species closely resembles *C.akisoides* based on the pronotum cordiform, but can be distinguished from the latter by the following characters (based on male): (1) emargination of outer margins of head above antennal base widely obtuse-angular (straight in *C.akisoides*); (2) distal part of metatibiae with a few golden yellow spines on inner side (with a row of golden yellow hairy row in *C.akisoides*); (3) parameres relatively narrow and long (wide and short in *C.akisoides*), widest at base, and narrowing toward apex nearly straight (basal 1/4 parallel, and then narrowing toward apex nearly straight in *C.akisoides*), distal part nearly straight in lateral view (weakly curved to ventral side in *C.akisoides*). This new species is also somewhat similar to *C.medvedevi* sp. nov. based on the distal part of metatibiae with a few golden yellow setae on inner side in male, it differs from the later by the following characters (based on male): (1) eyes not protruding beyond contour of head (slightly protruding beyond contour of head in *C.medvedevi* sp. nov.); (2) pronotum cordiform (transverse, subcordiform in *C.medvedevi* sp. nov.), anterior and posterior angles rectangular (nearly rectangular in *C.medvedevi* sp. nov.); (3) distal part of protibiae distinctly thick (protibiae gradually widened toward apex in *C.medvedevi* sp. nov.); (4) parameres relatively narrow and long (wide and short in *C.medvedevi* sp. nov.), widest at base, and narrowing toward apex nearly straight (basal 1/3 parallel, and then narrowing toward apex nearly straight in *C.medvedevi* sp. nov.), distal part nearly straight in lateral view (weakly curved to ventral side in *C.medvedevi* sp. nov.).

#### Distribution.

China: Guizhou.

#### Etymology.

The species name is derived from the type locality Bijie.

#### Description.

Body black, weakly shiny; legs shiny.

**Male. *Head*.** Apical maxillary palpomere triangular, covered with moderately dense and long setae. Anterior margin of labrum emarginate, lateral margins parallel. Anterior margin of epistoma emarginate, nearly straight at middle; surface flat, matte, inconspicuous punctured. Frontoepistomal suture shallow and arcuate. Dorsal surface of head flat, matte, sparsely and finely granulated. Genal margins arcuately converging forwards, densely and shallowly punctures merging into shallow wrinkles. Emargination of outer margins of head above antennal base widely obtuse-angular. Eyes transverse, not protruding beyond contour of head, distance between outer margins of eyes represent the widest of head; height 0.53 mm, width 0.17 mm from lateral view, respectively (height 3.1× width; 2.5× if the height and width rounded to one decimal place). Temples arcuately narrowing backwards, weakly granulated. Antennae slender and long, with the last segment reaching beyond pronotal base; basal part of antennomere I invisible in dorsal view; antennomeres II–VII cylindrical, thicker at apex, II shortest, III longest, V–VI equal in length, longer than IV and shorter than VII; antennomeres VIII–X nearly spherical; antennomere XI sharped-oval.

***Prothorax*.** Pronotum cordiform, widest at middle, 1.4× wide as long, 1.8× wide as head, ratio of width at anterior margin to middle and base 8: 12: 10; anterior margin deeply emarginate, beaded laterally; lateral margins weakly “S” curved, entirely beaded and smooth; posterior margin bisinuate, beaded laterally; anterior angles rectangular and protruding forwards, posterior angles rectangular; surface matte, central convex, lateral sides weakly depressed along lateral margins, with shallowly and rounded depressions in sides of central, moderately depressed near posterior angles in sides of base, longitudinal median line smooth and inconspicuous; shallowly, sparsely, and finely punctate in central part, near posterior margin, lateral margins, and lateral sides of anterior margin with wrinkly punctures, sparsely and finely granulated. Prothoracic hypomera depressed, densely and shallowly wrinkled in longitudinal, with sparse and tiny granules. Prosternal process sharply sloping downwards behind procoxae, apex blunt in lateral view.

***Pterothorax*.** Elytra oval, widest near middle, 1.3× long as wide, 1.4× wide as pronotum; base nearly as wide as pronotal base; dorsal surface matte, weakly convex, declivity sharply sloping downwards; humeral carinae smooth, with very sparse, smooth and large tubercles, sparse and fine granules, sparse, coarse and shallow wrinkles between humeral carinae; each elytron with two carinae between suture and humeral carina, the second carina inconspicuous; declivity with sparse and short setae, slightly convex along suture; surface of epipleuron matte, edge relatively wide; pseudopleuron much wider than epipleuron, surface matte, with sparse and inconspicuous granules, edge thin and elevated, reaching sutural angle. Scutellum triangular, covered by pronotum.

***Abdomen*.** Apex of ventrite 1 flat in middle; ventrites 1, 2, and anterior part of ventrite 3 rough, with moderately dense and long setae, and inconspicuous granules, densely, shallowly, and finely wrinkled at sides; posterior part of ventrite 3 smooth, shallowly punctate; ventrite 4 smooth, shallowly and finely punctate; last ventrite smooth, apex with sparse, fine punctures and short setae, apical margin widely rounded.

***Legs*.** Slender and long. Femora claviform, mesofemora slightly longer than profemora, and shorter than metafemora. Protibiae weakly curved, distal part distinctly thick; mesotibiae nearly straight, metatibiae straight, both gradually widened toward apex; distal part of metatibiae with a few golden yellow setae on inner side. Ventral surface of pro- and mesotarsomeres I–IV, and metatarsomeres I–III with undeveloped hairy tuft at apex.

***Aedeagus*.** Length 1.7 mm, width 0.5 mm. Parameres length 0.6 mm, width 0.4 mm. Parameres relatively narrow and long, widest at base, and narrowing toward apex nearly straight, distal part nearly straight in lateral view.

**Female.** Unknown.

#### Measurements.

Body length: ♂ 11.1 mm, width: ♂ 5.9 mm.

### ﻿ Colasia helenae

Taxon classificationAnimaliaColeopteraTenebrionidae

(Medvedev, 2007) comb. nov.

3A52627B-FFE3-5ECC-A5F2-9EBC00779440

Figs 7–10 , 33

Belousovia helenae Medvedev, 2007: 159; Ren et al. 2016: 328; Nabozhenko and Chigray 2020: 285.

#### Type material

**(studied). *Holotype***: ♂ (ZIN), CH, W Yunnan, SSW Liuku / 25 41 31 N / 98 47 16 E / H = 3000 m, 21.05.2006 / *Belousov* & *Kabak* leg. // Holotypus / Belousovia / helenae G.Medvedev // ZOOLOGICAL / INSTITUTE RAS / ST. PETERSBURG.

#### Additional material.

**China**: 4♂, 7♀ (HBUM), 2008-VII-25 / Liuku Town, [Lushui City], Yunnan / Ji-Shan XU leg. / Hebei University Museum.

#### Distribution.

China: Yunnan.

### ﻿ Colasia kabaki intermedia

Taxon classificationAnimaliaColeopteraTenebrionidae

(Medvedev, 2007) comb. nov.

47CC839E-2FCB-59ED-8BCA-DC641E6E9236

Figs 11–14 , 33

Belousovia kabaki intermedia Medvedev, 2007: 164; Ren et al. 2016: 331; Nabozhenko and Chigray 2020: 285.

#### Type material

**(studied). *Holotype***: ♂ (ZIN), CH, Yunnan, N Baoshan / 25 29 28 N / 99 05 35 E / 25 29 38 N / 99 04 51 E / 2790–3370 m, 09.05.2006 / *Belousov* & *Kabak* leg. // Holotypus / Belousovia kabaki / intermedia G. Medvedev // ZOOLOGICAL / INSTITUTE RAS / ST. PETERSBURG.

#### Distribution.

China: Yunnan.

### ﻿ Colasia kabaki kabaki

Taxon classificationAnimaliaColeopteraTenebrionidae

(Medvedev, 2007) comb. nov.

D77FCF15-E6D0-590F-9EFB-A78F4F3C878C

Figs 15–18 , 19 , 23 , 33

Belousovia kabaki kabaki Medvedev, 2007: 162; Ren et al. 2016: 330; Nabozhenko and Chigray 2020: 285.

#### Type material

**(studied). *Holotype***: ♂ (ZIN), CH, Yunnan, N Baoshan / 25 28 54 N / 99 05 05 E / H = 3200 m, 10.05.2006 / *Belousov* & *Kabak* leg. // Holotypus / Belousovia / kabaki G.Medvedev // ZOOLOGICAL / INSTITUTE RAS / ST. PETERSBURG.

#### Additional material.

**China**: 5♂, 3♀ (HBUM), 2008-VII-19 / Baoshan City, Yunnan / Ji-Shan XU et al. leg. / Hebei University Museum; 4♂, 1♀ (HBUM), 2009-IV-18 / Dapoqing, [Xieyangfeng Peak], Cang Shan, [Dali City], Yunnan, 2400 m / Zi-Zhong YANG leg. / Museum of China West Normal University; 1♀ (HBUM), 2009-VIII-22 / Dapoqing, Cang Shan, Yunnan, 2400 m / Ye ZHAO & Kui-Chang ZHANG leg. / Museum of China West Normal University; 3♂, 3♀ (HBUM), 2011-VI-20 / Dapoqing, Cang Shan, Yunnan / 2700 m, Wu-Bang WANG leg. / Biological Science Museum, Dali University; 8♂, 7♀ (HBUM), 2009-V-6 / 1986 Huoshaodi [Burned Blank of forest fires in 1986, refers to Dapoqing], Cang Shan, 2700 m / Ye ZHAO & Kui-Chang ZHANG leg. / Museum of China West Normal University; 1♂ (HBUM), 2009-VIII-7-9 / Ailao Shan, Jingdong County, Yunnan / Ji-Shan XU & Li-Xiang ZHANG leg. / Hebei University Museum // 24°32'30.3"N / 101°01'35.9"E / 2450 m / Hebei University Museum.

#### Distribution.

China: Yunnan.

#### Remarks.

Eye height 0.52 mm, width 0.17 mm from lateral view (height 3.1× width; 2.5× if the height and width are rounded to one decimal place).

### ﻿ Colasia medvedevi sp. nov.

Taxon classificationAnimaliaColeopteraTenebrionidae

8F9D5B20-38A3-57B6-AB75-443C09E915E2

https://zoobank.org/56F2B086-3BB0-4597-B8B5-D06614111873

Figs 20 , 24 , 25 , 33

Colasia akisioides sensu Medvedev, 2001: 110, 123, 145, 220, 243, 328, figs 68, 144, 254, 255, 873, 990, 1323.

#### Type material.

***Holotype***: ♂ (HBUM), 2006-VII-14 / Junzi Shan, Shizong County, Yunnan / Ben-Yong MAO et al. leg. / Hebei University Museum. ***Paratypes***: 2♂, 3♀ (HBUM), 2006-VII-14 / Junzi Shan, Shizong County, Yunnan / Ben-Yong MAO et al. leg. / Hebei University Museum; 1♂ (ZIN), China. Prov. Yunnan. Vallis flumin. Soling-ho. [Longchuan River, Yuanmou County (Wang 2022: 4)] // Coll. N. Skopin // Coll. G. Hauser // *Colasiaakisioides* Koch. Det. N.Skopin, 1977.

**Figures 7–18.**
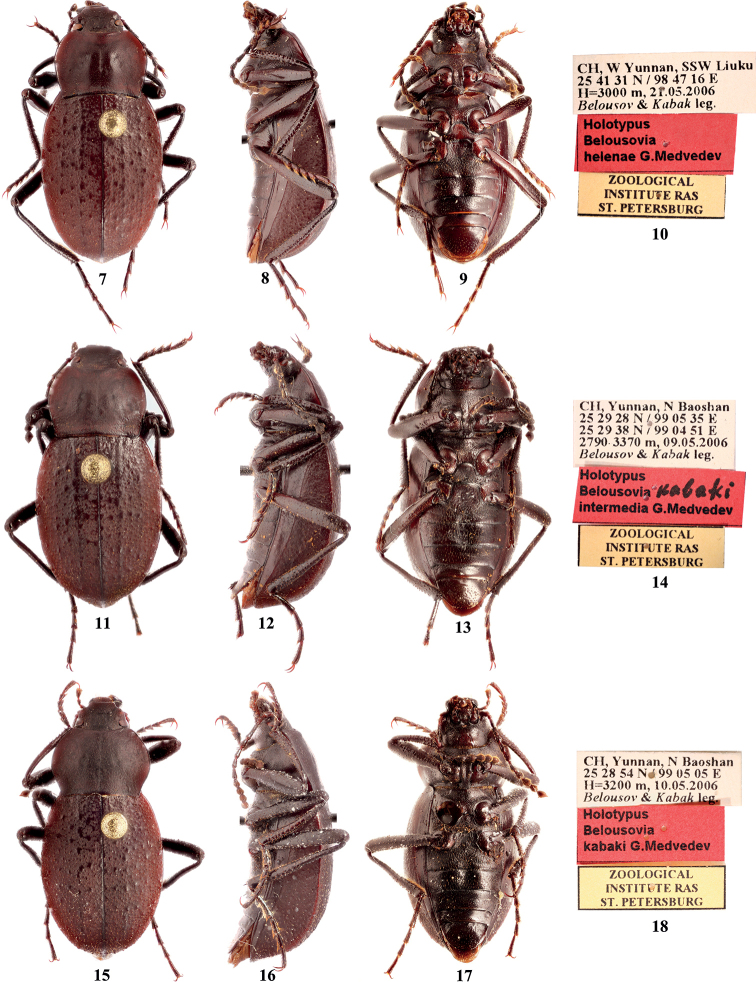
Habitus and type labels of **7–10***Colasiahelenae* (Medvedev) male, holotype **11–14***C.kabakiintermedia* (Medvedev) male, holotype, and **15–18***C.kabakikabaki* (Medvedev) male, holotype **7, 11, 15** dorsal views **8, 12, 16** lateral views **9, 13, 17** ventral views (photographs by Ivan Chigray).

#### Diagnosis.

This new species closely resembles *C.helenae*, *C.kabakiintermedia*, and *C.kabakikabaki* based on the pronotum transverse, subcordiform, but can be distinguished from the last three taxa by the following characters (based on male): (1) distal part of metatibiae with a few golden yellow spines and setae on inner side (with a row of golden yellow hairy brush in *C.helenae*, *C.kabakiintermedia*, and *C.kabakikabaki*); (2) elytral surface more wrinkled; (3) lateral margins of pronotum distinctly arcuate from middle to base (nearly straight in *C.helenae*, and *C.kabakiintermedia*); (4) basal 1/3 of parameres parallel, and then narrowing toward apex nearly straight (parameres widest at base, and narrowing toward apex nearly straight in *C.helenae*, and *C.kabakikabaki*). This new species is also similar to *C.bijica* sp. nov. based on the distal part of metatibiae with a few golden yellow setae on inner side in male, the differences between them see diagnosis of *C.bijica* sp. nov.

#### Distribution.

China: Yunnan.

#### Etymology.

The species name is derived from the name of Prof. Gleb Sergeevich Medvedev, in memory of his outstanding contribution to the knowledge of the tribe Blaptini of the tenebrionid beetles.

#### Description.

Body black, weakly shiny; legs shiny.

**Male. *Head*.** Apical maxillary palpomere triangular, covered with moderately dense and long setae. Anterior margin of labrum slightly emarginate, lateral margins parallel. Anterior margin of epistoma slightly emarginate; surface flat, matte, shallowly punctate. Frontoepistomal suture shallow and arcuate. Dorsal surface of head flat, matte, sparsely and finely granulated. Genal margins arcuately converging forwards, densely and shallowly punctures merged into short wrinkles. Emargination of outer margins of head above antennal base widely obtuse-angular. Eyes transverse, slightly protruding beyond contour of head, distance between outer margins of eyes represent the widest of head; height 0.52 mm, width 0.19 mm from lateral view, respectively (height 2.7× width; 2.5× if the height and width are rounded to one decimal place). Temples arcuately narrowing backwards, sparsely granulated. Antennae slender and long, with the last segment reaching beyond pronotal base; basal part of antennomere I invisible in dorsal view; antennomeres II–VII cylindrical, thicker at apex, II very short, III very long, V–VI equal in length, slightly longer than IV and shorter than VII; VIII–X nearly spherical; XI sharped-oval.

***Prothorax*.** Pronotum transverse, subcordiform, widest at middle, 1.5× wide as long, 1.9× wide as head, ratio of width at anterior margin to middle and base 7: 11: 8; anterior margin deeply emarginate, beaded laterally; lateral margins weakly “S” curved, entirely beaded and smooth; posterior margin straight at middle, beaded laterally; anterior angles nearly rectangular and protruding forwards, posterior angles nearly rectangular; surface matte, central convex, lateral sides weakly depressed along lateral margins, with shallowly and rounded depressions in sides of central, moderately depressed near posterior angles in sides of base, longitudinal median line smooth and weak; shallowly, sparsely, and finely punctate in central part, near posterior margin, lateral margins, and lateral sides of anterior margin with wrinkly punctures, sparsely and finely granulated. Prothoracic hypomera depressed, densely and shallowly wrinkled in longitudinal, with sparse and tiny granules. Prosternal process sharply sloping downwards behind procoxae, apex blunt in lateral view.

**Figure 19.**
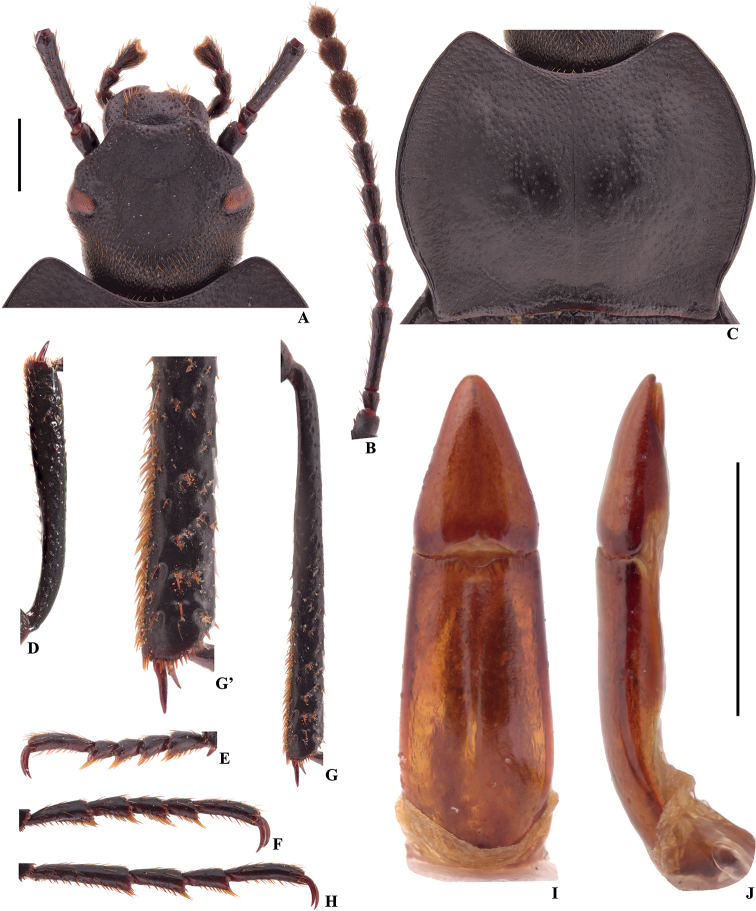
Characters of *Colasiakabakikabaki* (Medvedev) (male) **A** head **B** antenna **C** pronotum **D** protibia **E** protarsus **F** mesotarsus **G** metatibia **G**’ distal part of metatibia **H** metatarsus **I, J** aedeagus in dorsal and lateral view, respectively. Scale bars: 1.0 mm.

***Pterothorax*.** Elytra oval, widest at middle, 1.3× long as wide, 1.3× wide as pronotum; base nearly as wide as pronotal base; dorsal surface matte, relatively flat, declivity sharply sloping downwards; humeral carinae smooth, with very sparse, smooth and large tubercles, sparse and fine granules, sparse, coarse and shallow wrinkles between humeral carinae; each elytron with two carinae between suture and humeral carina, the second carina inconspicuous; declivity with sparse and short setae; surface of epipleuron matte, edge relatively wide; pseudopleuron much wider than epipleuron, surface matte, with sparse and inconspicuous granules, edge thin and elevated, reaching sutural angle. Scutellum triangular, covered by pronotum.

**Figure 20.**
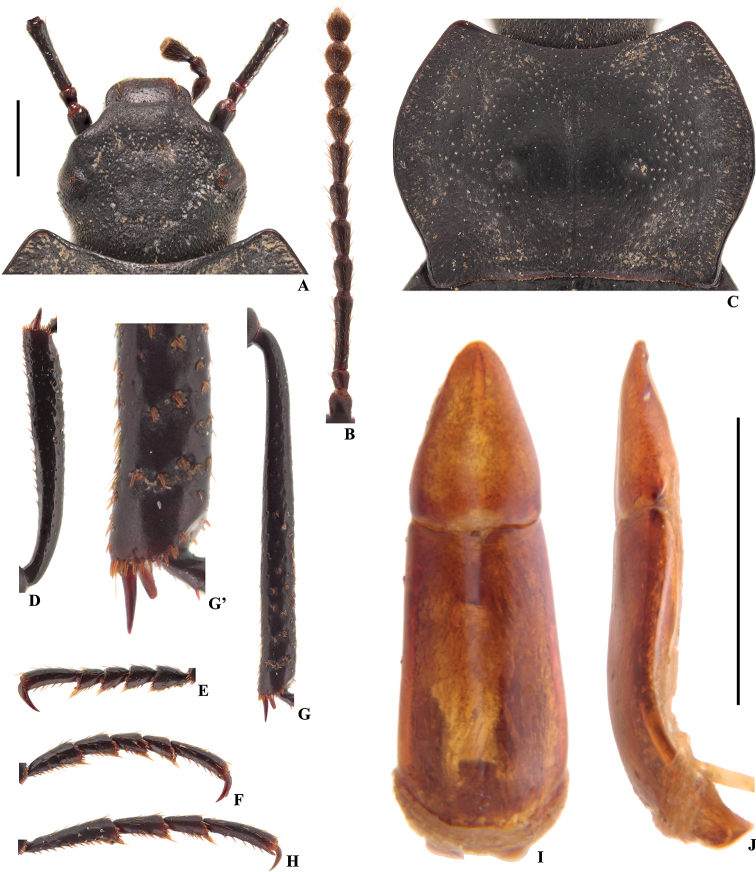
Characters of *Colasiamedvedevi* sp. nov., male, holotype **A** head **B** antenna **C** pronotum **D** protibia **E** protarsus **F** mesotarsus **G** metatibia **G**’ distal part of metatibia **H** metatarsus **I, J** aedeagus in dorsal and lateral view, respectively. Scale bars: 1.0 mm.

***Abdomen*.** Apex of ventrite 1, and base of ventrite 2 flat in middle; ventrites 1, 2, and anterior part of ventrite 3 rough, with moderately dense and long setae, and inconspicuous granules, densely, shallowly, and finely wrinkled at sides; posterior part of ventrite 3 smooth, shallowly punctate; ventrite 4 smooth, shallowly and finely punctate; last ventrite smooth, with sparse, fine punctures and short setae, apical margin widely rounded.

***Legs*.** Slender and long. Femora claviform, mesofemora slightly longer than profemora, and shorter than metafemora. Protibiae weakly curved, mesotibiae nearly straight, metatibiae straight, both gradually widened toward apex; distal part of metatibiae with a few golden yellow setae on inner side. Ventral surface of pro- and mesotarsomeres I–IV, and metatarsomeres I–III with undeveloped hairy tuft at apex.

***Aedeagus*.** Length 1.3 mm, width 0.6 mm. Parameres length 0.7 mm, width 0.5 mm. Parameres relatively wide and short, widest at base, basal 1/3 parallel, and then narrowing toward apex nearly straight, distal part weakly curved to ventral side in lateral view.

**Female.** Antennae not reaching pronotal base; elytra wider and convex; abdominal ventrites 1 and 2 convex; inner side of metatibiae without golden yellow setae; other characters similar to male.

#### Measurements.

Body length: ♂ 12.8–13.5 mm, ♀ 13.0–14.0 mm; width: ♂ 6.2–6.5 mm, ♀ 7.2–7.5 mm.

### ﻿ Colasia pilosa sp. nov.

Taxon classificationAnimaliaColeopteraTenebrionidae

6D751AAA-A261-5AEB-BD8A-FBFE0D32E7B5

https://zoobank.org/DD8788CB-7E54-428F-9835-C0D1C1376B55

**Figure 21.**
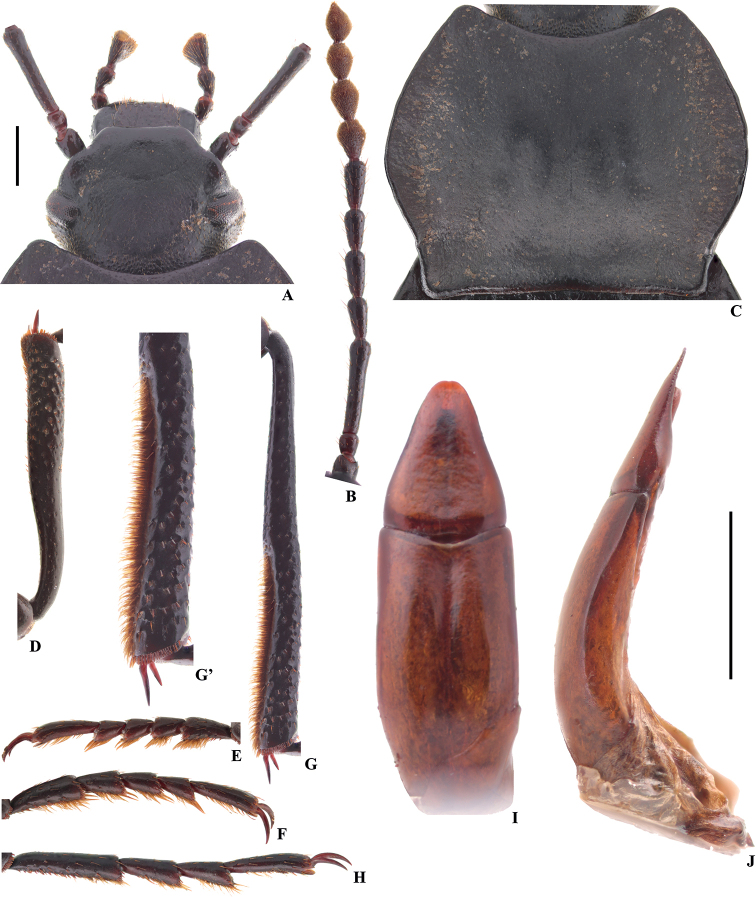
Characters of *Colasiapilosa* sp. nov., male, holotype **A** head **B** antenna **C** pronotum **D** protibia **E** protarsus **F** mesotarsus **G** metatibia **G**’ distal part of metatibia **H** metatarsi **I, J** aedeagus in dorsal and lateral view, respectively. Scale bars: 1.0 mm.

Figs 21 , 26 , 27 , 30–32 , 33

#### Type material.

***Holotype***: ♂ (HBUM), 2016.V.6 / Dajian Shan, Dawei Shan Nature Reserve, Pingbian County, Yunnan, 2100 m / Lu QIU leg. / Mianyang Normal University, MYNU. ***Paratypes***: 1♀ (HBUM), 2016.V.6 / Dajian Shan, Dawei Shan Nature Reserve, Pingbian County, Yunnan, 2100 m / Lu QIU leg. / Mianyang Normal University, MYNU; 1♀ (MYNU), 2015.V.20 / Dajian Shan, Dawei Shan Nature Reserve, Pingbian County, Yunnan, 2100 m / Jian-Yue QIU leg. / Mianyang Normal University, MYNU; 1♀ (MYNU), 2016.X.27 / Dajian Shan, Dawei Shan Nature Reserve, Pingbian County, Yunnan, 2100 m / Gui-Qiang HUANG & Yan-Chao WANG leg. / Mianyang Normal University, MYNU; 1♂, 2♀ (MYNU), 2018.V.25-27 / Dajian Shan, Dawei Shan Nature Reserve, Pingbian County, Yunnan, 2100 m / Hao XU & Jian-Yue QIU leg. / Mianyang Normal University, MYNU; 1♂ (MYNU), 2021.V.29-VI.3 / Dajian Shan, Dawei Shan Nature Reserve, Pingbian County, Yunnan, 2050 m / Hao XU, Xin-Yuan ZHANG & Rui-Ying LIU leg. / Mianyang Normal University, MYNU; 3♂ (CTLH), 2022.V.28 / Dawei Shan Yakou, Pingbian County, Honghe Zhou [equivalent to City], Yunnan, 2050 m / Tian-Long HE & Zi-Dan XU leg.; 1♀ (CTLH), 2022.V.29 / Dawei Shan Yakou, Pingbian County, Honghe Zhou [equivalent to City], Yunnan, 2050 m / Tian-Long HE & Zi-Dan XU leg.; 1♂ (HBUM), 2022-V-28 / Dawei Shan Yakou, Pingbian County, Yunnan / 2050 m, Tian-Long HE & Zi-Dan XU leg. / Hebei University Museum; 1♀ (HBUM), 2022-V-29 / Dawei Shan Yakou, Pingbian County, Yunnan / 2050 m, Tian-Long HE & Zi-Dan XU leg. / Hebei University Museum; 2♂, 3♀ (HBUM), 2010-V-22 / Dawei Shan, Pingbian County, Yunnan / Chang-Chin CHEN leg. / Hebei University Museum; 1♀ (HBUM), 2010-V-20 / Dawei Shan, Pingbian County, Yunnan / 2100 m, Chang-Chin CHEN leg. / Hebei University Museum; 1♀ (HBUM), 2014-V-9-10 / Dawei Shan, Pingbian County, Yunnan / Xiao-Yu ZHU leg. / Hebei University Museum; 1♂, 1♀ (HBUM), 2015-VI-20-21 / Dawei Shan, Pingbian County, Yunnan / Zi-Zhong YANG leg. / Institute of Entomoceutics Research, Dali University; 2♂, 4♀ (HBUM), 2005-IV-25 / Pingbian County, Yunnan / Ben-Yong MAO leg. / Hebei University Museum.

**Figures 22–27.**
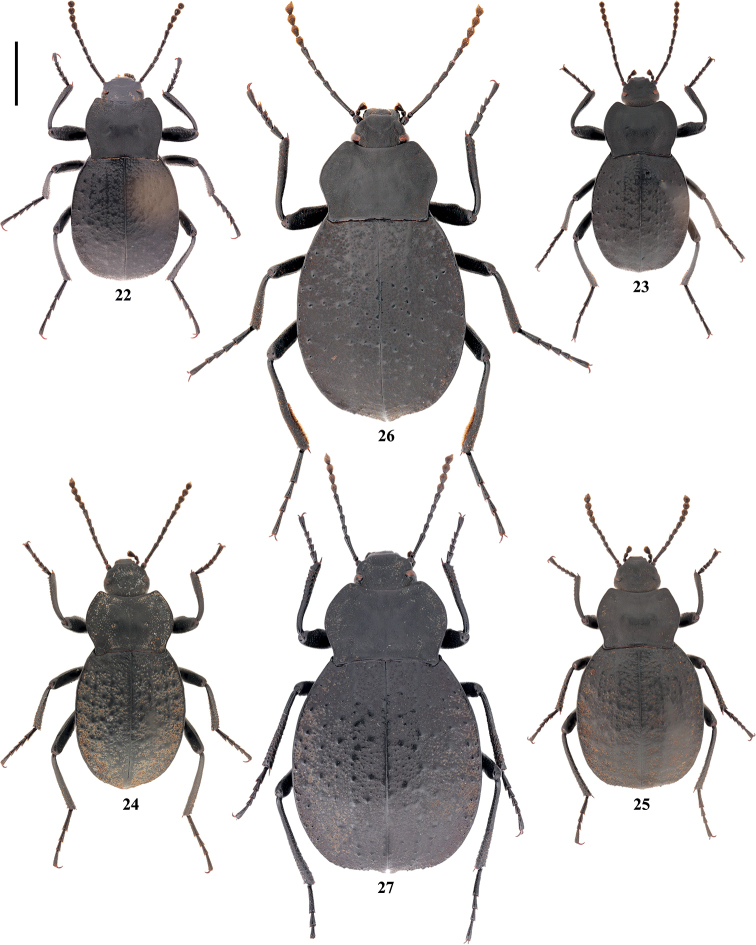
Habitus of **22***Colasiabijica* sp. nov. male, holotype **23***C.kabakikabaki* (Medvedev) male **24, 25***C.medvedevi* sp. nov. **24** male, holotype **25** female, paratype, and **26, 27***C.pilosa* sp. nov. **26** male, holotype **27** female, paratype. Scale bar: 3.5 mm.

#### Diagnosis.

This new species is obviously different from other species in the following characters (based on male): body large size, length 15.5–17.5 mm (vs. 11.1–13.8 mm in other species); inner side of metatibiae with a row of well-developed densely hairy brush from middle to apex; ventral surface of pro- and mesotarsomeres I–IV, and metatarsomeres I–III with well-developed hairy brush from middle to apex; parameres laminar in dorsal view.

#### Distribution.

China: Yunnan.

#### Etymology.

The species name is derived from the dense hairy brush of male metatibiae.

#### Description.

Body black, weakly shiny.

**Male. *Head*.** Apical maxillary palpomere triangular, covered with moderately dense and long setae. Anterior margin of labrum slightly emarginate, lateral margins weakly arcuate. Anterior margin of epistoma slightly emarginate; surface flat, matte, shallowly punctate. Frontoepistomal suture shallow and arcuate. Dorsal surface of head flat, matte, sparsely granulated at sides. Genal margins arcuately converging forwards, densely and shallowly punctate. Emargination of outer margins of head above antennal base widely obtuse-angular. Eyes transverse, protruding beyond contour of head, distance between outer margins of eyes represent the widest of head; height 0.80 mm, width 0.26 mm from lateral view, respectively (height 3.1× width; 2.7× if the height and width are rounded to one decimal place). Temples arcuately narrowing backwards, sparsely granulated. Antennae slender and long, with the last segment reaching beyond pronotal base; basal part of antennomere I invisible in dorsal view; antennomeres II–VII cylindrical, thicker at apex, II very short, III very long, IV and VI subequal in length, V and VII subequal in length; VIII–X nearly spherical; XI sharped-oval.

***Prothorax*.** Pronotum transverse, subcordiform, widest at middle, 1.5× wide as long, 1.9× wide as head, ratio of width at anterior margin to middle and base 6: 10: 8; anterior margin deeply emarginate, beaded laterally; lateral margins weakly “S” curved, entirely beaded and smooth; posterior margin straight at middle, beaded laterally; anterior angles nearly rectangular and protruding forwards, posterior angles nearly rectangular; surface matte, central convex, lateral sides weakly depressed along lateral margins, with moderately depressions in middle of sides and near posterior angles in sides of base, sometimes shallowly and transversely depressed before posterior margin in middle, longitudinal median line inconspicuous; shallowly, sparsely, and finely punctate in central part, near posterior margin, lateral margins, and anterior margin with wrinkly punctures, sparsely and finely granulated. Prothoracic hypomera depressed, densely and shallowly wrinkled in longitudinal, with sparse and tiny granules. Prosternal process sharply sloping downwards behind procoxae, apex blunt in lateral view.

***Pterothorax*.** Elytra oval, widest at middle, 1.2× long as wide, 1.4× wide as pronotum; base wider than pronotal base; dorsal surface matte, relatively flat, declivity sharply sloping downwards; humeral carinae smooth, with very sparse, smooth and large tubercles, sparse and fine granules, sparse, coarse and shallow wrinkles between humeral carinae; each elytron with two carinae between suture and humeral carina, the second carina inconspicuous; declivity with sparse and short setae, slightly convex along suture; surface of epipleuron matte, edge relatively wide; pseudopleuron much wider than epipleuron, surface matte, sparsely granulated, edge thin, reaching sutural angle. Scutellum triangular, covered by pronotum.

**Figures 28–32.**
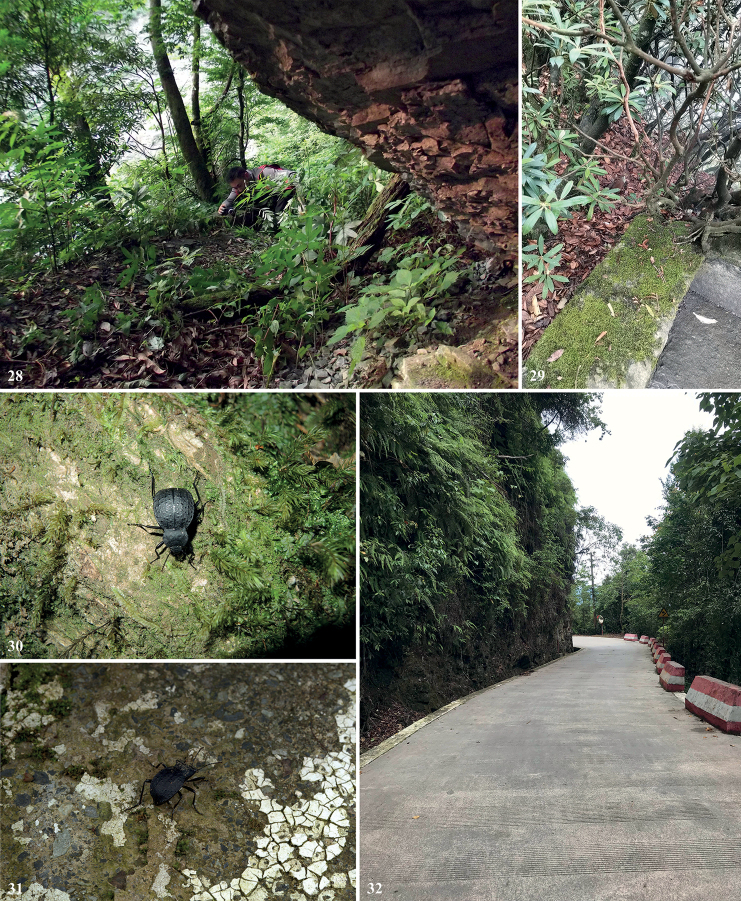
Habitats of *Colasiaakisoides* Koch **28** photograph by Ri-Xin Jiang **29** photograph by Tian-Xuan Gu. *C.pilosa* sp. nov. **30** female, photograph by Lu Qiu **31** male, photograph by Tian-Long He **32** photograph by Tian-Long He.

***Abdomen*.** Apex of ventrites 1 and 2 weakly depressed or flat in middle; ventrites 1,2, and anterior part of ventrite 3 rough, with moderately dense and long setae, and inconspicuous granules, densely, shallowly, and finely wrinkled at sides; posterior part of ventrite 3 smooth, shallowly punctate; ventrite 4 smooth, shallowly and finely punctate; last ventrite smooth, with sparse, fine punctures and short setae, apical margin widely rounded.

***Legs*.** Slender and long. Femora claviform, mesofemora slightly longer than profemora, and shorter than metafemora. Protibiae and metatibiae weakly curved, mesotibiae straight, both gradually widened toward apex; inner side of metatibiae with a row of densely golden yellow hairy row from middle to apex. Ventral surface of pro- and mesotarsomeres I–IV, and metatarsomeres I–III with dense hairy brush from middle to apex.

***Aedeagus*.** Length 2.8 mm, width 0.9 mm. Parameres length 1.0 mm, width 0.7 mm. Parameres relatively wide and short, widest at base, and narrowing toward apex nearly straight, distal part straight in lateral view.

**Female.** Antennae not reaching pronotal base; elytra wider and more convex; abdominal ventrites 1 and 2 convex; inner side of metatibiae without golden yellow hairy row; ventral surface of tarsi without hairy brush; other characters similar to male.

#### Measurements.

Body length: ♂ 15.5–17.5 mm, ♀ 16.0–18.0 mm; width: ♂ 8.5–9.5 mm, ♀ 9.5–10.6 mm.

**Figure 33.**
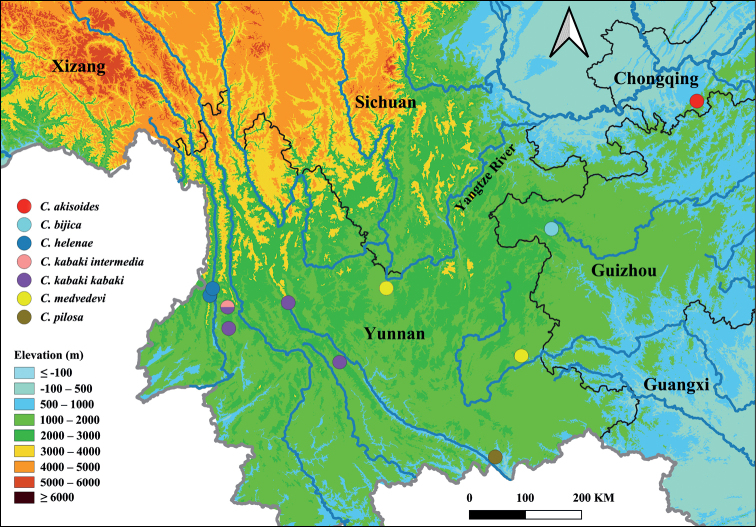
Distribution of the species of the revised genus *Colasia* (known to occur only in the south of the Yangtze River).

### ﻿Key to species of the revised genus *Colasia* Koch, 1965 (based on males)

**Table:** 

1	Body length 15.5–17.5 mm; inner side of metatibiae with a row of well-developed densely hairy brush from middle to apex (Fig. 21G’); ventral surface of pro- and mesotarsomeres I–IV, and metatarsomeres I–III with well-developed hairy brush from middle to apex (Fig. 21E, F, H)	***C.pilosa* sp. nov.**
–	Body length 11.1–13.8 mm; inner side of metatibiae with or without a row of hairy brush (Figs 5G’, 6G’); ventral surface of pro- and mesotarsomeres I–IV, and metatarsomeres I–III with less-developed hairy brush from middle to apex (Fig. 19E, F, H; Medvedev 2007: fig. 116) or with undeveloped hairy tuft at apex (Fig. 6E, F, H)	**2**
2	Eyes not protruding beyond contour of head (Fig. 6A); pronotum strongly cordiform, anterior and posterior angles rectangular (Fig. 6C)	**3**
–	Eyes slightly protruding beyond contour of head (Fig. 20A); pronotum transverse, weakly cordiform, anterior and posterior angles weakly obtuse or subrectangular (Fig. 20C)	**4**
3	Lateral margins of head straight above antennal base (Fig. 5A); distal part of metatibiae with a row of setae on inner side (Fig. 5G’); parameres relatively wide and short, basal 1/3 parallel, and then narrowing toward apex nearly straight, distal part weakly curved to ventral side in lateral view (Fig. 5I, J)	***C.akisoides* Koch, 1965**
–	Lateral margins of head widely obtuse-angled above antennal base (Fig. 6A); distal part of metatibiae with a few setae on inner side (Fig. 6G’); parameres relatively narrow and long, widest at base, and narrowing toward apex nearly straight, distal part nearly straight in lateral view (Fig. 6I, J)	***C.bijica* sp. nov.**
4	Distal part of metatibiae with a few setae on inner side (Fig. 20G’); lateral margins of pronotum distinctly arcuate from middle to base (Fig. 20C); basal 1/3 of parameres parallel, and then narrowing toward apex nearly straight (Fig. 20I, J)	***C.medvedevi* sp. nov.**
–	Distal part of metatibiae with a row of setae on inner side (Fig. 19G’); lateral margins of pronotum distinctly arcuate (Fig. 20C) or nearly straight from middle to base (Medvedev 2007: fig. 125); parameres widest at base, and narrowing toward apex nearly straight; or basal 1/3 of parameres parallel, and then narrowing toward apex nearly straight (Fig. 19I, J; Medvedev 2007: figs 103, 119, 128)	**5**
5	Basal 1/3 of parameres parallel, and then narrowing toward apex nearly straight (Medvedev 2007: fig. 128)	***C.kabakiintermedia* (Medvedev, 2007)**
–	Parameres widest at base, and narrowing toward apex nearly straight (Fig. 19I, J; Medvedev 2007: figs 103, 119)	**6**
6	Lateral margins of pronotum nearly straight from middle to base (Medvedev 2007: fig. 101)	***C.helenae* (Medvedev, 2007)**
–	Lateral margins of pronotum distinctly arcuate from middle to base (Fig. 19C)	***C.kabakikabaki* (Medvedev, 2007)**

## Supplementary Material

XML Treatment for Colasia

XML Treatment for Colasia akisoides

XML Treatment for Colasia bijica

XML Treatment for Colasia helenae

XML Treatment for Colasia kabaki intermedia

XML Treatment for Colasia kabaki kabaki

XML Treatment for Colasia medvedevi

XML Treatment for Colasia pilosa

## References

[B1] Bezděk A Král D Sládeček FXJ (2015) Oniticellus (Liatongus) boucomonti Balthasar, 1932 (Coleoptera: Scarabaeidae: Scarabaeinae: Oniticellini)–clarification of its taxonomic status by lectotype designation. Zootaxa 3974(1): 145–147. 10.11646/zootaxa.3974.1.13 26249892

[B2] Bouchard P Bousquet Y Aalbu RL Alonso-Zarazaga MA Merkl O Davie AE (2021) Review of genus-group names in the family Tenebrionidae (Insecta, Coleoptera). ZooKeys 1050: 1–633. 10.3897/zookeys.1050.64217 34385881 PMC8328949

[B3] Ge SX Hu SJ Shi HL Han FY Li MJ Ren LL (2021) The first record of the genus *Belenois* (Lepidoptera: Pieridae) from China. Biodiversity Data Journal 9: e61332. 10.3897/BDJ.9.e61332 PMC7835196 33519265

[B4] Kamiński MJ Kanda K Lumen R Smith AD Iwan D (2019) Molecular phylogeny of Pedinini (Coleoptera, Tenebrionidae) and its implications for higher-level classification. Zoological Journal of the Linnean Society 185: 77–97. 10.1093/zoolinnean/zly033

[B5] Kamiński MJ Lumen R Kanda K Iwan D Johnston MA Kergoat GJ Bouchard P Bai XL Li XM Ren GD Smith AD (2021) Reevaluation of Blapimorpha and Opatrinae: addressing a major phylogeny-classification gap in darkling beetles (Coleoptera: Tenebrionidae: Blaptinae). Systematic Entomology 46(1): 140–156. 10.1111/syen.12453

[B6] Kataev BM Liang HB (2015) Taxonomic review of Chinese species of ground beetles of the subgenus Pseudoophonus (genus *Harpalus*) (Coleoptera: Carabidae). Zootaxa 3920(1): 1–39. 10.11646/zootaxa.3920.1.1 25781237

[B7] Koch C (1965) Sur les types de Fairmaire des tribus Blaptini et Platyscelini conservés au Muséum de Paris (Col. Tenebrionidae). Annales de la Société Entomologique de France 1: 125–135. [Nouvelle Série]

[B8] Leach WE (1815) Entomology: 57–172. In: Brewster D (Ed.) Brewster’s Edinburgh Encyclopedia (Vol. IX) [part I]. W. Blackwood, J. Waugh, etc., Edinburgh, 764 pp.

[B9] Li XM Bai XL Kergoat GJ Pan Z Ren GD (2021) Phylogenetics, historical biogeography and molecular species delimitation of *Gnaptorina* Reitter (Coleoptera: Tenebrionidae: Blaptini). Systematic Entomology 46(1): 239–251. 10.1111/syen.12459

[B10] Löbl I Nabozhenko M Merkl O (2008) Tribe Blaptini Leach, 1815: 219–238. In: Löbl I, Smetana A (Eds) Catalogue of Palaearctic Coleoptera (Vol. 5). Tenebrionoidea. Apollo Books, Stenstrup, 670 pp.

[B11] Medvedev GS (1998) To the knowledge of the tenebrionid beetles of the tribe Blaptini (Coleoptera, Tenebrionidae) of eastern part of the Tibet plateau. Entomological Review 78: 79–111. [in Russian, Entomologicheskoe Obozrenie 77: 171–208]

[B12] Medvedev GS (2001) Evolution and system of darkling beetles of the tribe Blaptini (Coleoptera, Tenebrionidae). Meetengs in memory of N.A. Kholodkovsky. Iss. 53. Russian Entomological Society Press, St.-Petersburg, 332 pp. [In Russian]

[B13] Medvedev GS (2007) A contribution to the taxonomy and morphology of the tribe Blaptini (Coleoptera, Tenebrionidae). Entomological Review 87(2): 181–214. [In Russian, Entomologicheskoe Obozrenie 86(1): 132–170] 10.1134/S0013873807020078

[B14] Medvedev GS (2009) Composition of the genera *Gnaptorina* Reitter and *Pseudognaptorina* Kaszab of the tribe Blaptini (Coleoptera, Tenebrionidae). Entomological Review 89(4): 451–461. [In Russian, Entomologicheskoe Obozrenie 88(2): 416–429] 10.1134/S0013873809040095

[B15] Medvedev GS Merkl O (2003) *Viettagonavietnamensis* gen. et sp. n. from Vietnam (Coleoptera, Tenebrionidae: Blaptini). Acta Zoologica Academiae Scientiarum Hungaricae 48 [2002]: 317–332.

[B16] Nabozhenko M Chigray I (2020) Tribe Blaptini Leach, 1815: 268–296. In: Iwan D, Löbl I (Eds) Catalogue of Palaearctic Coleoptera (Vol. 5). Tenebrionoidea. Revised and Updated Second Edition. Koninklijke Brill NV, Leiden-Boston, 945 pp. 10.1163/9789004434998

[B17] Ren GD Ba YB Liu HY Niu YP Zhu XC Li Z Shi AM (2016) Fauna Sinica. Insecta (Vol. 63). Coleoptera. Tenebrionidae (I). Science Press, Beijing, 532 pp. [In Chinese, with English abstract]

[B18] Skopin NG (1960) Material on the morphology and ecology of larvae of the tribe Blaptini (Coleoptera, Tenebrionidae). Trudy instituta zoologii Akademii nauk Kazakhskoy SSR 11: 36–71. [In Russian]

[B19] Wang FL (2022) Three new species of genus *Anomala* Samouelle from Yunnan, China (Coleoptera: Scarabaeidae, Rutelinae). Faunitaxys 10(56): 1–9. 10.57800/faunitaxys-10(56)

